# The dynamics of mergers and acquisitions: ancestry as the seminal determinant

**DOI:** 10.1098/rspa.2014.0370

**Published:** 2014-11-08

**Authors:** Eduardo Viegas, Stuart P. Cockburn, Henrik J. Jensen, Geoffrey B. West

**Affiliations:** 1 Centre for Complexity Science and Department of Mathematics, Imperial College London, London SW7 2AZ, UK; 2 PricewaterhouseCoopers LLP, 7 More London Riverside, London SE1 2RT, UK; 3 Santa Fe Institute, 1399 Hyde Park Road, Santa Fe, NM 87501, USA

**Keywords:** evolutionary dynamics, ecosystems, economics

## Abstract

Understanding the fundamental mechanisms behind the complex landscape of corporate mergers and acquisitions is of crucial importance to economies across the world. Adapting ideas from the fields of complexity and evolutionary dynamics to analyse business ecosystems, we show here that ancestry, i.e. the cumulative sum of historical mergers across all ancestors, is the key characteristic to company mergers and acquisitions. We verify this by comparing an agent-based model to an extensive range of business data, covering the period from the 1830s to the present day and a range of industries and geographies. This seemingly universal mechanism leads to imbalanced business ecosystems, with the emergence of a few very large, but sluggish ‘too big to fail’ entities, and very small, niche entities, thereby creating a paradigm where a configuration akin to effective oligopoly or monopoly is a likely outcome for free market systems.

## 1. Introduction

Mergers and acquisitions of firms and companies are fundamental activities that dynamically shape the business world. However, there is a lack of consensus and full understanding of the drivers and benefits to businesses arising from those activities within conventional financial studies [1–3]. These tend to centre on financial and economic data which primarily give a snapshot of the emergent state of markets at a specific time. In contrast, we show here that ancestry, defined as the cumulative number of mergers from all acquired entities, is the major determinant and indicator shaping markets; we find a ‘snowball’ effect to be active, whereby entities with larger ancestries are found to be more likely to merge or acquire again. Our study employs ideas inspired from the fields of complex networks and evolutionary dynamics to analyse business ecosystems [4–6], with particular focus on diversity reduction.

Specifically, we translate the important distinction between phenotype and genotype to a business context: the former being associated with externally observable attributes, influenced by a combination of genetic, environmental and developmental factors, whereas the latter refers to the more fundamental, underlying genetic instructions that encapsulate the design for these attributes [7]. Whereas financial and economic data represent the phenotype, ancestry is analogous to the genotype, as it encodes the relevant past evolutionary processes that determine the probability for an institution to become the acquirer of another business. This is demonstrated by testing the ancestry mechanism against data from several countries, industries and timescales using a variant of a preferential attachment agent-based model. This seemingly universal mechanism also leads to a sizewise bimodality in the distribution of resulting entities, and to an imbalanced ecosystem mostly consisting of a few very large, ‘too big to fail’ entities, and small, niche entities, thereby creating a paradigm where a configuration akin to effective oligopoly or monopoly is a likely outcome for free market systems.

## 2. Ancestry data analysis

### (a) Methods to predict mergers: ancestry versus balance sheet size

There are a range of factors that may contribute to the merger and acquisition process—such as the regulatory environment, balance sheet size of a business, potential synergies across different agents, value of a brand, etc. However, we show that ancestry is the primary characteristic that captures the essential merger dynamics, and therefore synthesizes all the above factors. To illustrate this point, we considered a ranking test, to study both ancestry and balance sheet size as merger predictors, using data from the US banking industry.

**Figure 1.**
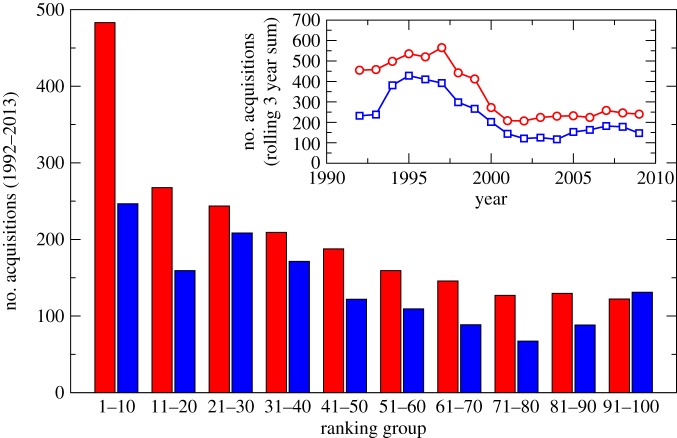
Comparison of ranking methods to predict merger activity for US banks: ancestry (red) versus balance sheet size (blue). US banks are ranked based on ancestry (red) or balance sheet size (blue). The bar chart shows the time-averaged number of mergers between banks (vertical axis) by ranking group (horizontal axis). Here, an increasing ranking group corresponds to a decrease in ancestor number or balance sheet size, as appropriate. For each ranking group, the number of acquisitions is averaged over successive 3 year periods, starting at each year between 1992 and 2013. The inset shows the ancestry (red) and balance sheet (blue) ranking methods as a function of time. Each data point shows the number of mergers that occurred within a 3 year window from the year indicated by its position on the horizontal axis; the curves therefore compare the acquisition numbers over time for the top 100 banks by ancestor number and the top 100 banks by balance sheet size.

In figure 1, US banks are ranked based on ancestry (red) or balance sheet size (blue). The bar chart shows the time-averaged number of mergers between banks (vertical axis) by ranking group (horizontal axis). Here, an increasing ranking group corresponds to a decrease in ancestor number or balance sheet size, as appropriate. For each ranking group, the number of acquisitions is averaged over a 3 year period, starting at each year between 1992 and 2013. As shown in figure 1, ranking banks by ancestry number led to monotonically decreasing merger activity from the high- to low-ranking groups, i.e. those banks ranked higher by ancestor number systematically partook in more mergers. Ranking instead by balance sheet size, we found that the number of mergers varied far less smoothly and predictably down the ranking groups, strongly indicating ancestry as a more consistent predictor for merger likelihood.

The inset of figure 1 considers the two ranking methods as a function of time. This is done by ranking the banks by either ancestry number or balance sheet size, taking the top 100 banks in each case. The red (upper) curve shows data for the top 100 banks when ranked by ancestry, and the blue (lower) curve shows data for the top 100 banks when ranked by balance sheet size. Each data point on the curve shows the number of mergers that occurred within a 3 year window from the year indicated by its position on the horizontal axis, for the group of banks represented. Together, the curves show that the top 100 group ranked by ancestry consistently underwent more mergers than the top 100 group ranked by balance sheet size, indicating that ancestor number also leads to a better ranking as a function as time.

The balance sheet of a company is, of course, a key measure of its financial state, and can be considered an important tool for predicting possible future acquisitions. Indeed, a number of studies indicate that larger entities are more likely to be acquirers [8]. However, there is a strong correlation between balance sheet size and ancestry, akin to phenotype and genotype respectively, so our finding that ancestry plays a fundamental role in predicting acquisitions is not necessarily inconsistent with traditional finance theory. On the contrary, our framework clarifies the relationship between the two: ancestry, representing the historical trajectory of a company, is the underlying seminal property that drives the dynamics of mergers and acquisitions between companies. In turn, this leads to changes to balance sheet size, which is a measure of the state of the company at a given time.

Therefore, we conclude that first, entities with many ancestors are generally more likely to be involved in merger activities, and second, that ranking by ancestry places the banks in a more accurate order, in terms of merger likelihood, than ranking by balance sheet size.

### (b) Ancestry data across different industries and geographies

**Figure 2.**
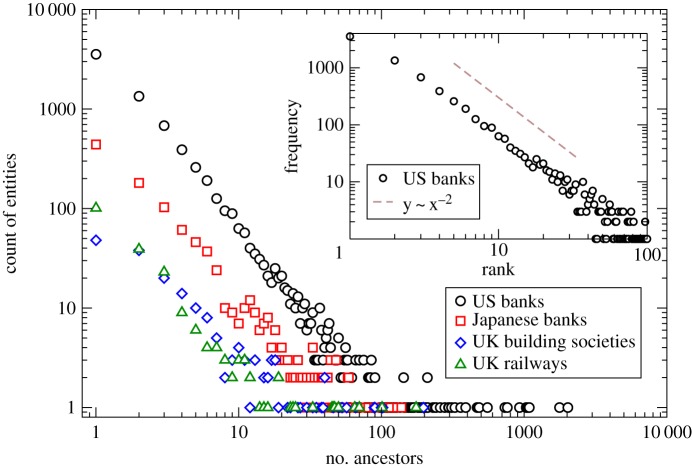
Structure of ancestry data. Log–log plot showing distributions of ancestry numbers across several industries and countries. Inset: Zipf plot for US banking ancestry data; the dashed line has a gradient of −2.

In figure 2, we consider the importance of ancestry to merger activities in greater detail using data for business ancestries across a range of different industries. These cover: US banks at national and state-levels, Japanese banks, UK building societies and UK railway companies, over various time periods spanning 1830–2012. The respective data sources were: the Federal Deposit Insurance Corporation Institutional Directory, the Japanese Banking Association (JBA) data directory, the UK Building Societies Association member files and data on UK railway companies contained in Awdry’s *Encyclopaedia of British Railway Companies* [9].

In each case, the observable of interest is the distribution of businesses by ancestry size, i.e. the distribution of businesses ranked by their number of ancestors on a cumulative basis across the entire time period under consideration. From figure 2, it is clear that there is a striking similarity between all the observed distributions, with each exhibiting power-law-like behaviours with similar slopes on the log–log scale shown.

## 3. Evolutionary dynamics

To isolate the underlying system dynamics, we develop a simple agent-based model for a market in which mergers are assumed to occur, and compare this initially with the US banking dataset.

### (a) Agent-based model

The basic aim of the agent-based model is to capture the ancestry-driven (i.e. genotype) merger dynamics that underlie observable financial data, the phenotype of this analogy. Our model is a variant of the classic preferential attachment model of Yule [10] and is closely related to Price’s cumulative advantage theory [11] and Simon’s stochastic process formulation [12]. Let us remark on how the dependence on ancestry relates to the preferential attachment concept [12,13] and cumulative advantage [14]. In relation to our study, these mechanisms are clearly distinct from each other. The term preferential attachment is used to describe a process where the new, and smaller, vertices express a preference towards attaching to larger vertices. Therefore, the behaviour of the agent—the new arriving vertex—is determined by features of another agent. In contrast, cumulative advantage is a process where an inherent characteristic of an agent drives the behaviour of that same agent. The dependence of the merger probability on ancestry is, in this sense, an example of ‘cumulative advantage’ and distinctly different from the preferential attachment mechanism.

The agents of the model are represented as a vector, **B**=[*B*_0_,*B*_1,…_], and undergo cycles of dynamics where mergers between agents occur with a probability that depends on how many ancestors, *n*_A_, each already has. Motivated by the Zipf-like distribution [15] of figure 2, which approximates a power-law with exponent −2, the probability of a merger for each agent is taken to be of the simple form

3.1 $$p_{\mathrm{merger}}=p{\left(1+n_{A}\right)}^{3/2}.$$

**Figure 3.**
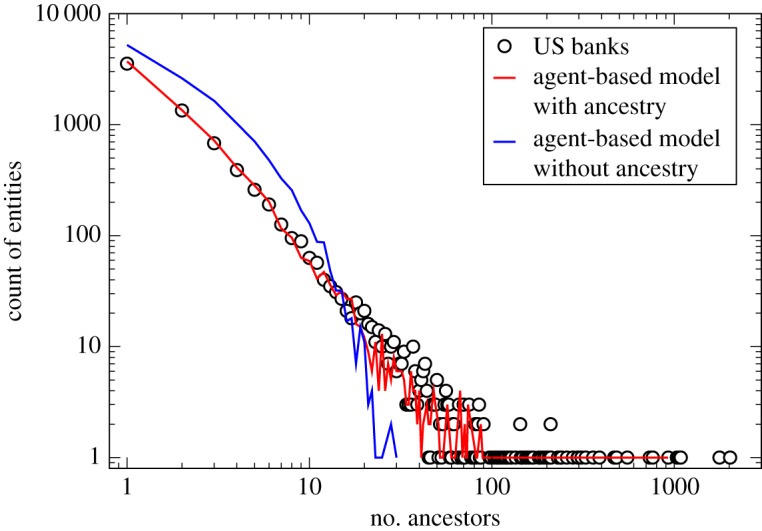
Importance of ancestry dependence. A comparison is shown between US banking ancestry data and model simulations with (red) and without (blue) an ancestry weighted merger probability. Clearly, the former leads to a far closer agreement with the US data.

Here, $p=1/{\left(1+n_{A}^{\max}\right)}^{3/2}$ is a constant, determined by the maximum potential ancestry number for a given dataset $n_{A}^{\max}=N_{\mathrm{initial}}-N_{\mathrm{final}}$, with *N*_initial_ and *N*_final_ the initial and final number of entities, respectively. Despite this choice, we note at this point that a large range of values for the parameter *p* in fact yield very similar results. In addition, note that setting *n*_A_ to zero in this equation would yield a purely random model, i.e. the random walk model of figure 3.

In each cycle, source banks are selected based on the ancestry-based probability of equation (3.1), after which each randomly selects a merger partner as a target; these partners then cease to exist in the live population, and the source bank adds the target bank’s ancestors to its own number of ancestors. The initial number of agents is obtained directly from the dataset being modelled, and each agent is deemed to have no ancestors on model initialization. Cycles continue until the total number of remaining agents is equal to the corresponding number in the final time input data. The model data shown are based on 1000 repeated simulations.

The first key point to highlight from the model is the non-stationary, evolving nature of the mergers and acquisitions process for companies, which is clearly demonstrated in figure 3; here, we compare the results of a single realization of the full model (red line) to the actual accumulated ancestry data for the US nationally (black circles), and contrast those with the results of a ‘random walk’ model (blue line), where in the latter all agents have equal, *time-independent*, probability, *p*, to merge (this corresponds to setting *n*_A_=0 in equation (3.1) so that *p*_merger_=*p*). The ‘random walk’ model leads to a notable deviation from the US data and a loss of the power-law structure of the ancestry distribution, whereas a merger probability that depends on the past history through ancestry size yields excellent agreement across the entire range. We conclude that ancestry size can be viewed as encoding how keen a company is to originate a merger, i.e. the ancestry record encodes a phenotypical trait.

**Figure 4.**
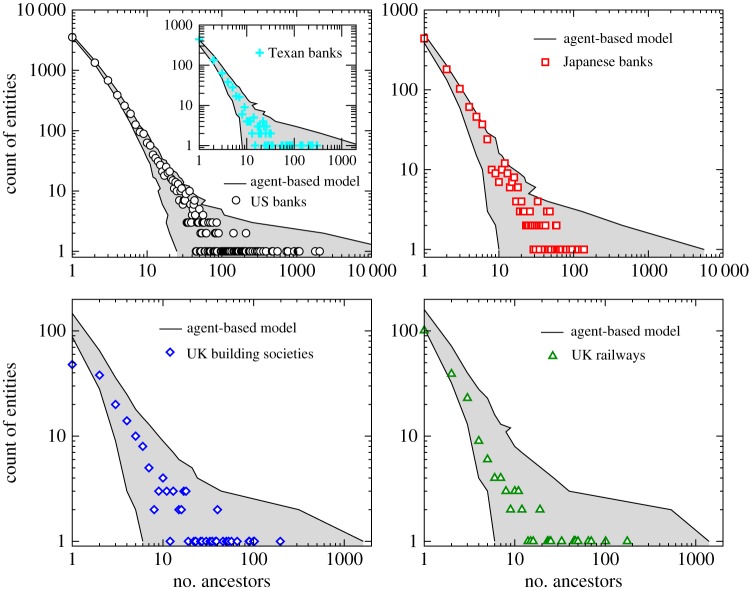
Model versus real-world data. Mergers data are shown for US banks (1970–2013), Japanese banks (1872–2013), UK building societies (1936–2012) and UK railway companies (late 1700s–1923). In each case, the model predictions are shown by the grey-shaded region, which indicates the range of predictions across 1000 simulations. The inset shows the subset of US data for banks within Texas.

To test the model further, we show in figure 4*a* detailed comparison of several additional sets of data: the US (national and state-specific; 1970–2013) and Japanese (1872–2013) banking sectors, and the UK building societies (1936–2012) and railway companies (late 1700s–1923). Given the very distinct markets, industries, and timelines covered by these datasets, the close agreement between the model and results suggest that these differences are of little consequence for the merger and acquisitions process over a long-term evolutionary period, and, importantly, that an ancestry-based model captures the fundamental and essential dynamics across these systems. It is important to note that the period considered encompasses two banking crisis in the US, as well as other financial crises and the war periods in the case of Japan and the UK datasets. It is likely, however, that significant changes in activity may exist within very short timespans. It is also interesting to note that the regulatory framework that exists in the USA is fragmented in nature, as different banks need to comply with regulations that can be federal banking statutes and/or state-specific laws. We can therefore consider the similarity between state and federal level as indicating a certain robustness to the ancestry mechanism. And indeed, the distinctive legislation, as well as time period of acquisitions, does not seem to bear any impact on the long-term evolutionary process.

Restricting the analysis to individual US states, we also find a very favourable agreement between the model predictions and data. This suggests that a kind of self-similarity exists between subsets of the systems (e.g. the individual states, in the US case) and the entire system (e.g. all US banks); an example from the largest banking state, Texas, is shown in figure 4 (upper left inset). The latter is particularly interesting, given the changing regulatory view of interstate mergers in the USA. The 1982 Garn-St. Germain Depository Institutions Act allowed interstate mergers and acquisitions only for failed banks [16]. This restriction persisted until 1994, after which interstate mergers were made legal for all banks through the Riegle–Neal Interstate Banking and Branching Efficiency Act. So, owing to legislative restrictions, most *intra*state mergers and acquisitions occurred before 1994, yet the observed dynamics appears to be insensitive to these legislative restrictions.

### (b) Mean field master equation

In addition to our agent-based model, we furthered our work by adopting the mean field theory approach. In essence, the approach aims to translate complex stochastic models—such as the agent based model described above—into a simpler deterministic model expected to capture some average behaviour of the agents through the development of a master equation. Given the inherent approximations necessary to make analytical progress, we would not expect the master equation to fully replicate the agent-based model results. Instead, the aim of this exercise is to provide an alternative method that can give an additional insight of the behaviour of the system.

An exact master equation for the probability density of the ancestry-based merger dynamics is difficult to establish. However, the algorithm of the agent-based model may be approximated by the following process. Assume we have *N* bins, and that at *t*=1 each bin contains 1 ball, i.e. *n*_A_=1 for all bins. At each time step, a bin is chosen to initiate a merger event with probability *p*_mer_(*n*_A_), which is equivalent to *p*_merger_ in equation (3.1). A merger event consists of choosing one of the other (non-empty) bins with uniform probability and moving the contents of the chosen bin to the originating bin. We let one timestep consist of one attempted merger event. This means that for times *t*>1, the ancestry variable can assume values: *n*_A_=0,1,2,…,*t*.

Introducing the probability distribution *P*(*n*_A_) as the mean field, ensemble probability to find a company with ancestry *n*_A_ at time *t*, the master equation for the above process is straightforward. We can distinguish the following three cases: *n*_A_=0,

3.2 $$P(0,t+1)=P(0,t)+\sum_{n=1}^{t}p_{\mathrm{mer}}(n)P(n,t)[1-P(0,t)].$$

*n*_A_=1,

3.3 $$P(1,t+1)=P(1,t)\left[1-p_{\mathrm{mer}}(1)\sum_{k=1}^{t}P(k,t)-\sum_{n=1}^{t}p_{\mathrm{mer}}(n)P(n,t)\right].$$

2<*n*_A_<*t*,

3.4 $$\begin{array}{rl} P(n_{A},t+1) & =P(n_{A},t)+\sum_{k=1}^{n_{A}-1}p_{\mathrm{mer}}(k)P(k,t)P(n_{A}-k,t)\\ & \;-p_{\mathrm{mer}}(n_{A})P(n_{A},t)\sum_{k=1}^{t}P(k,t)\\ & \;-\sum_{k=1}^{t}p_{\mathrm{mer}}(k)P(k,t)P(n_{A},t). \end{array}$$

**Figure 5.**
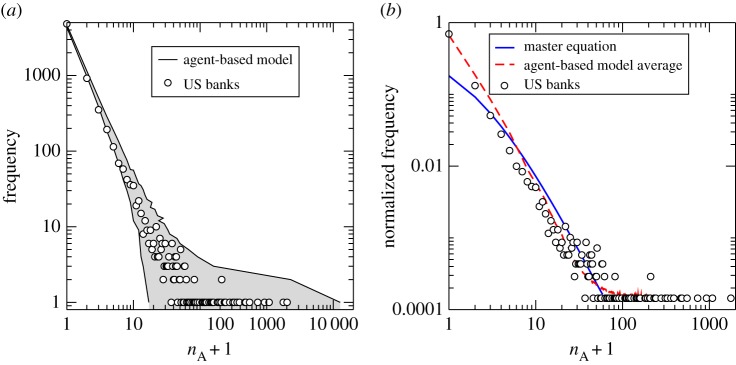
Comparison between the master equation, agent-based model and real data from the surviving population in the US banking sector. (*a*) The range of ancestry data obtained from the agent-based model over 1000 simulations (grey-shaded area), compared with that for the live population within the real US banking dataset (black circles). (*b*) A comparison with the master equation result. Both plots are shown as functions of the shifted ancestry variable *n*_A_+1.

A comparison between the results of the master equation and the surviving population of the agent-based model described in §3*a*, each relative to the real US banking data is shown in figure 5. When comparing the results of figure 5, given the simplifying assumptions, in particular, the fact that correlations between the states of the agents are neglected, we consider that the master equation provides a reasonable approximation of the results of the agent-based model and actual data. However, we note that agreement is not consistent across all ancestry numbers, in particular for small ancestor numbers.

## 4. Perspectives for businesses

Given that mergers and acquisitions can be understood primarily in terms of ancestry rather than as a result of temporary local financial conditions, the question arises as to how financially beneficial such processes are to individual businesses.

### (a) Growth through mergers

**Figure 6.**
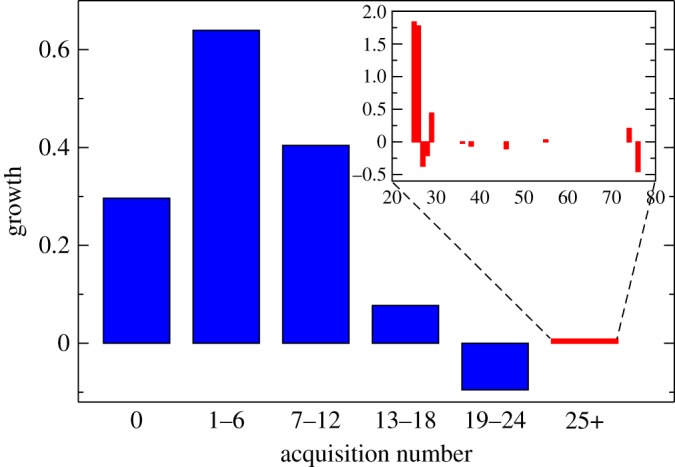
Real business organic growth. The chart shows the actual, organic growth of entities within each acquisition grouping, excluding the effects of GDP growth and balance sheet aggregation from mergers between US banks from 1992 to 2013. For each of the surviving banks in 2013, we aggregated the balance sheets of each of their ancestors in 1992, and indexed by the GDP. As a result, the real organic growth is derived from the ratio between the balance sheets of surviving entities as at 2013, and those of their respective ancestors (GDP linked and aggregated) as at 1992. The scale is relative to GDP such that values >0, =0 and <0 indicate growth greater than, tracking, and less than GDP, respectively. The red bar in the main plot shows the average over those banks with ancestries >25; the inset shows a refined view of growth for these largest banks, by number of acquisitions. Note that in averaging the data shown in the inset to arrive at the >25 bucket shown in the main plot, the average is weighted by balance sheet size.

In figure 6, we show an analysis of the growth of the US banks as a function of acquisition number. This clearly shows that growth levels do not increase, on average, for entities having undergone numerous mergers, but instead that they grow at a rate close to that of the GDP. This levelling off of growth is reminiscent of deterministic growth typically seen in biological organisms. Indeed, companies with a small number of acquisitions (which are typically smaller and younger) systematically manifest higher growth on average (although note that this growth is modest, at less than a percent above GDP). Despite the aggregate picture that the largest entities do not realize growth from acquisitions, of course individual businesses can experience pronounced growth, as shown in the inset to figure 6.

### (b) Dominance of largest entities

Although real growth is at best modest for individual companies, we now explore the changes in total assets across different bank size classes, ranked by centiles, to reveal some surprising consequences of ancestry-based merger dynamics upon a market as a whole.

**Figure 7.**
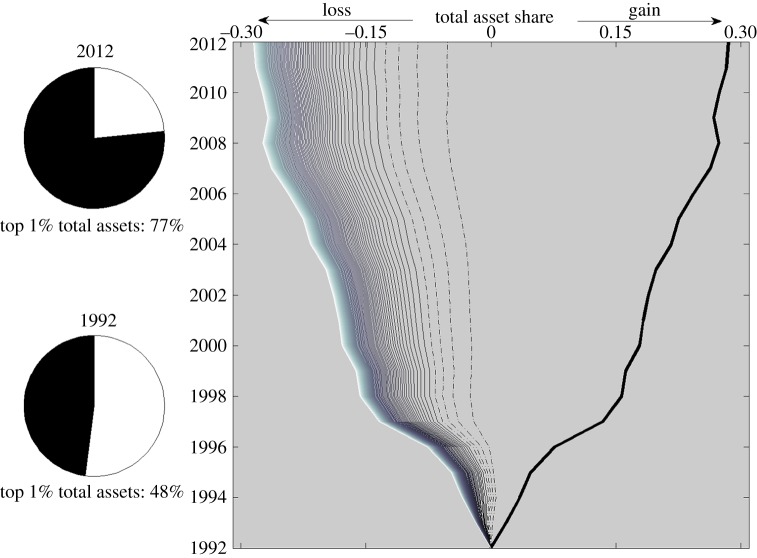
Sizewise bimodality in US banks. The plot shows the change in the market share of US banks over time, measured by growth in fractional balance sheet size. The banks are ranked into percentiles by proportion of total balance sheet size, with the top 5% isolated for clarity in the plot: the rightmost, thick black line is the top 1%; the next 4 percentiles are shown by dot-dashed lines. Moving to the left, the remaining lines represent each of the remaining percentiles. The second–100th percentile lines are plotted in a cumulative sense, to illustrate that the total loss from all of these market sections is equal to the growth in the largest 1% of the market. The gap between each of the second–100th percentile lines, therefore indicates the size of the loss for each percentile. The plot shows that the gap, and so loss, is largest for the second–10th percentile range, and decreases for subsequent percentiles, illustrating the mid-sized part of the market has suffered the largest loss by 2012.

Focusing upon the US banking sector, figure 7 shows the change in total assets share based on balance sheet data from 1992 to 2012. Figure 7 shows a clear increase in the total assets of the largest 1% of banks (thick black line), partnered with a reduction in the total assets held by entities from all other percentiles. The increase in total assets is almost exclusively concentrated within the largest 1% of banks, whereas the corresponding reduction in total assets comes mainly from the percentiles 2–10, with the remaining higher percentiles (and so smaller entities), contributing far less. This shows that it is the total assets of the top 2–10% banks by size, or mid-sized section of the market, that are depleted most drastically in order to fuel the growth of the largest banks.

The mid-sized section of the market therefore sees the greatest reduction in market share. This happens because the ancestry-driven mechanism underlying bank mergers and acquisitions affects this part of the market in two ways: (i) it leads to a power-law distribution of ancestries, and therefore many fewer agents in this size range and (ii) although greater in number than the largest entities, the mid-size agents are far less aggressive acquirers than the largest entities. Ultimately, this situation effectively leads to a bimodal population, in which only either very large entities or very small entities survive.

## 5. Conclusion

Our findings provide three key insights of broader relevance to free markets systems: first, traditional financial analyses would benefit from using non-financial as well as financial data in assessing market dynamics in the same manner that biological systems benefit from harmonious analysis of interactions between genotype and phenotype. Second, from a regulatory perspective, our findings suggest that legislation designed to ensure healthy competition, and ultimately benefit consumers and the broader economy (http://www.competition-commission.org.uk/about-us. accessed 4 February 2014), is not solely focused on the acquisitions among large entities, but should also protect the organic growth of smaller, rapidly expanding enterprises. Finally, while counterintuitive, the findings that mergers do not contribute to an enhancement of growth for entities with very large ancestries naturally opens a debate as to whether, as in the world of biology, companies’ growth follows a sigmoidal curve, meaning that there is a natural size limit beyond which growth is no longer advantageous.

We speculate that—similar to the natural world—companies are highly complex organisms that require close integration of enormous number of constituent units in order to work efficiently. In living organisms, delivery of energy to cells is optimized by vascular systems that set the pace of physiological processes as scaling functions of the size of the organisms [17, 18]. As a result, life cannot be sustained in sizes beyond those scaling laws. In contrast, within economic systems, corporates may be able to survive and grow to sizes above optimal levels. However, such growth may result in potentially large inefficiencies, as for example the observed trend in Scandinavian research institutions and the National Health Service in the UK, where the number of support staff has increased at a notably faster rate than the increase in number of front line delivery staff [19].

## Supplementary Material

Comments on the raw data files

## Supplementary Material

US Banks raw ancestry data

## Supplementary Material

Japanese Banks raw ancestry data

## Supplementary Material

Building Societies raw ancestry data

## Supplementary Material

UK Rail Companies raw ancestry data

## Supplementary Material

Extended data

## References

[1] Kwan S Eisenbeis A 1999 Mergers of publicly traded banking organizations revisited. Econ. Rev. Fed. Reserve Bank Atlanta Q4, 26–37

[2] Madura J Wiant KJ 1994 Long-term valuation effects of bank acquisitions. J. Bank. Fin. 18, 1135–1154 (doi:10.1016/0378-4266(94)00064-6)

[3] Toyne MF Tripp JD 1998 Interstate bank mergers and their impact on shareholder returns: evidence from the 1990s. Q. J. Bus. Econ. 37, 48–58

[4] Ueno H Mizuno T Takayasu M 2007 Analysis of Japanese banks historical tree diagram. Physica A 383, 164–168 (doi:10.1016/j.physa.2007.04.098)

[5] Viegas E Takayasu M Miura W Tamura K Ohnishi T Takayasu H Jensen HJ 2013 Ecosystems perspective on financial networks: diagnostic tools. Complexity 19, 22–36 (doi:10.1002/cplx.21452)

[6] Haldane AG May RM 2011 Systemic risk in banking ecosystems. Nature 469, 351–355 (doi:10.1038/nature09659) 21248842 10.1038/nature09659

[7] Jablonka E Lamb MJ 2005 Evolution in four dimensions: genetic, epigenetic, behavioral, and symbolic variation in the history of life. A Bradford Book. Cambridge, MA: MIT Press

[8] Beccalli E Frantz P 2013 The determinants of mergers and acquisitions in banking. J. Financ. Serv. Res. 43, 265–291 (doi:10.1007/s10693-012-0138-y)

[9] Awdry C 1990 Encyclopaedia of British railway companies. Wellingborough, UK: Patrick Stephens Limited

[10] Yule GU 1925 A mathematical theory of evolution, based on the conclusions of Dr. J. C. Willis, F.R.S. Phil. Trans. R. Soc. Lond. B 213, 402–410 (doi:10.1098/rstb.1925.0002)

[11] de Solla Price DJ 1976 A general theory of bibliometric and other cumulative advantage processes. J. Am. Soc. Inf. Sci. 27, 292–306 (doi:10.1002/asi.4630270505)

[12] Simon HA 1955 On a class of skew distribution functions. Biometrika 42, 425–440 (doi:10.1093/biomet/42.3-4.425)

[13] Barabasi A-L Albert R 1999 Emergence of scaling in random networks. Science 286, 509–512 (doi:10.1126/science.286.5439.509) 10521342 10.1126/science.286.5439.509

[14] Newman MEJ 2010 Networks: an introduction. New York, NY: Oxford University Press

[15] Zipf GK 1942 The unity of nature, least-action, and natural social science. Sociometry 5, 48–62 (doi:10.2307/2784953)

[16] Sherman M A short history of financial deregulation in the United States. Centre for Economic and Policy Research. See http://www.cepr.net/index.php/publications/reports/a-short-history-of-financial-deregulation-in-the-united-states/ (accessed 2 September 2014)

[17] Bettencourt LMA Lobo J Helbing D Kuhnert C West GB 2007 Growth, innovation, scaling, and the pace of life in cities. Proc. Natl Acad. Sci. USA 104, 7301–7306 (doi:10.1073/pnas.0610172104) 17438298 10.1073/pnas.0610172104 PMC1852329

[18] West GB Brown JH Enquist BJ 1997 A general model for the origin of allometric scaling laws in biology. Science 276, 122–126 (doi:10.1126/science.276.5309.122) 9082983 10.1126/science.276.5309.122

[19] Jamtveit B Jettestuen E Mathiesen J 2009 Scaling properties of European research units. Proc. Natl Acad. Sci. USA 106, 13160–13163 (doi:10.1073/pnas.0903190106) 19625626 10.1073/pnas.0903190106 PMC2714277

