# Supplementary material for: The dynamics of mergers and acquisitions: ancestry as the seminal determinant
Source: Proc Math Phys Eng Sci. 2014 Nov 8;470(2171):20140370. doi: 10.1098/rspa.2014.0370 (PMC4197467; doi:10.1098/rspa.2014.0370)
Supplement: Comments on the raw data files [file rspa20140370Supp1.pdf]

[Website]
Version 1.2

INFORMATION OF SUPPLEMENTAL ELECTRONIC MATERIAL – SOURCE DATA

US BANKS

DATA FILENAME

‘USBanksRawAncestryData.mat’ (MatLab file)

DATA FORMAT

US bank data held by the Federal Deposit Insurance Corporation (FDIC) can be downloaded as a spreadsheet (‘INSTITUTIONS2.CSV’) directly from the following FDIC website link:

http://www2.fdic.gov/idasp/main.asp

Several attributes that appear as columns in the FDIC spreadsheet (‘CERT’, ‘ACTIVE’, ‘EFFDATE’, ‘NEWCERT’, and ‘STNAME’) are contained within the US bank Matlab file named ‘USBanksRawAncestryData.mat’, included here.

The attribute ‘MISSINGCERT’, which also appears in the US bank Matlab file, was manually created through direct enquires to the directorate database.

SPECIFIC DATA MATTERS

Within the Matlab file ‘USBanksRawAncestryData.mat’, each attribute is stored as a vector. Definitions of the relevant attributes can be downloaded from the above link (spreadsheet ‘INSTITUTIONS2_DEFINITIONS.CSV’), however a brief summary follows.

The elements within a given row across all the Matlab vectors (except ‘MISSINGCERT’) collectively describe an event. Looking at a given row within each of the vectors, the element in:

- ‘CERT’ corresponds to the source entity, or, in other words, the acquired entity
- ‘NEWCERT’ is the target entity, or acquirer
- ‘EFFDATE’ corresponds to the date of the event
- ‘STNAME’ is the state name
- ‘ACTIVE’ is a flag indicating whether the bank was active at the date of download

In addition, ‘MISSINGCERT’ corresponds to banks that have left the FDIC.

Note that events with no ‘NEWCERT’’ number do not relate to mergers and acquisitions and were discarded. A bank’s sole rename was not regarded as a merger activity. Banks that dissolved or liquidated before any merger were not considered as part of the initial population.

JAPANESE BANKS

DATA FILENAME

'JapaneseBanksRawAncestryData.mat' (MatLab file)

DATA FORMAT

The file contains five vectors extracted from the Japanese Banking Association (JBA) website, which we divide into two groups:

(1) The population: The vector named 'ListofAllBanks' contains all banks listed in the JBA's database.

(2) The events: This group is made up of four vectors of equal length. The vector named 'Event' contains the descriptions of all events, with 'Year' containing the year of each event. For all events, the 'Source' vector gives the acquired entities and the 'Target' vector contains the acquirers. Across these vectors, the four elements in a given row collectively describe a merger event. E.g., the 1st row in 'Year' gives the year of the 1st event; the 1st row in 'Event' gives the type of event that occurred, while the 1st rows of 'Target' and 'Source' give the acquiring and acquired entities, respectively, for the 1st event.

SPECIFIC DATA MATTERS

A bank's renaming was not regarded as a merger activity. As a result, those events were not counted in the ancestries. Splits were excluded from the analysis, and regarded as new entities. Banks that dissolved before any merger were not considered as part of the initial population (banks that dissolved are represented by 'NaN' values in the 'Target' vector).

UK RAILWAY COMPANIES

DATA FILENAME

'RailCompaniesRawAncestryData.mat' (MatLab file)

DATA FORMAT

Each row contains only one populated column where the name of a Railway Company is included. Names in the first column correspond to all surviving entities before implementation of the Railway Act 1921 (year 1923) (known as "*Grouping*"). The second column corresponds to those companies that were acquired by the names in the first column. The acquirer for a given non-zero row in the second column, can be read off as the Railway Company appearing in the last (i.e. lowest) non-empty row of the first column, appearing above the row in question. The third to eighth columns all follow the same rationale described for the second column, where acquirers are located at the last non-empty rows of the previous (i.e. next left) columns.

SPECIFIC DATA MATTERS

The few existing Joint-Ownerships that remained unsolved before "*Grouping"* were allocated the company with which records indicate there was the longest relationship. For example, the Lancashire Union Railway became jointly owned by the Lancashire & Yorkshire Railway ("L&YR") and the London & North Western Railway ("L&NWR") in 1883, and passed to the London Midland & Scottish Railway ("LMS") at *Grouping*. The company in this example was allocated to the L&YR given that both they already had a jointly operation on part of the track since 1868. Any impact on this allocation is restricted to the tail of the

ancestry distribution and can be regarded as irrelevant for our analysis.

UK BUILDING SOCIETIES

DATA FILENAME

'BuildingSocietiesRawAncestryData.mat' (MatLab file)

DATA FORMAT

The structure is similar to that of the UK Railway Companies data. Each row contains only one populated column where the name of a Building Society is included. Names in the first column corresponds to all surviving entities as at the end of June 2013 (note details described below in Specific Data Matters). The second column corresponds to the societies that were acquired by the names in the first column, where the acquirer corresponds to the last non-empty row of the first column. The third to eighth columns follow the same rationale as described for the second column, where each acquirer for an entity in a given column is again located at the last non-empty row of the previous column.

SPECIFIC DATA MATTERS

Acquisitions of the societies by a Banking Group (i.e. entities that are not building societies) were not counted as a merger activity, since the ancestry of the Banking Group would be required. For example, Halifax was converted into a public limited company in 1997, subsequently merging with the Bank of Scotland. The ancestry analysis does not consider this merger.

One of the Building Societies – the Sun – was dissolved in 1992. The ancestry up to the date of dissolution was included in the analysis.

A number of societies have changed names during the period. Name changes do not form part of the ancestry analysis. As a result, the data file contains only one name for each given entity.
