# Supplementary material for: The dynamics of mergers and acquisitions: ancestry as the seminal determinant
Source: Proc Math Phys Eng Sci. 2014 Nov 8;470(2171):20140370. doi: 10.1098/rspa.2014.0370 (PMC4197467; doi:10.1098/rspa.2014.0370)
Supplement: Extended data [file rspa20140370Supp6.pdf]

The dynamics of mergers and acquisitions: ancestry as the seminal determinant - Extended data

Eduardo Viegas,[1] Stuart P. Cockburn,[2] Henrik J. Jensen,[1] and Geoffrey B. West[3,1]

[1] *Complexity & Networks Group and Department of Mathematics, Imperial College London, SW7 2AZ, United Kingdom*

[2] *PricewaterhouseCoopers LLP, 7 More London Riverside, London, SE1 2RT*

[3] *Santa Fe Institute, 1399 Hyde Park Road, Santa Fe, NM 87501, United States*

(Dated: May 7, 2014)

I. EXTENDED DATA

A. Stability testing - sensitivity to initial condition

The model data presented in the main paper is taken from simulations carried out using the agent based model described in Section 3a of the main paper. The model is always set up so that each agent in the initial population has an ancestry value $n_{\mathrm{A}} = 0$. In this Section, we describe tests undertaken using the US banking data that examine how sensitive the model is to the choice of initial condition.

The US banking simulation data shown in the main paper covers the period from 1970 to 2013. In the tests described in this Section, we consider modelling the mergers and acquisitions processes that occurred between 1934 to 1969, ending immediately before the simulations of the main paper began and therefore generating an initial condition. The starting year was chosen because the Banking Act of 1933 (often referred to as the Glass-Steagall Act) fundamentally reformed the US banking systems, including the establishment of the Federal Deposit Insurance Corporation (FDIC). The aim of these tests is to generate an alternative initial condition, such that all Banks are no longer initially considered to have zero ancestors at 1970, and examine how this effects the ancestry distribution produced for 2013.

Based on Supplementary Table 1, to generate an initial condition as at 1970, we therefore start our simulations from 1934 with a population of 19637 banks. As a cross-check on this value, it is useful to compare the data gleaned from the Federal Reserve Annual Report to data held by FDIC. We note initially that the FDIC population as at 01/01/1970 is in fact 18194 banks, therefore there is a difference of 4036 between the FDIC and Federal Reserve data that must be understood and reconciled. This difference turns out to be due mainly to two factors: (i) Banks that were regulated by the OTS, and not part of the Federal Reserve numbers ($\sim$ 3600); (ii) Banks that were not part of the Federal Reserve system but which became so after 1970 ($\sim$ 300).

In terms of the simulations of this Section, the initial condition at 1970 is a population of 27631 banks, broken down as follows: 13473 banks are deemed to have zero ancestors (4036 as the reconciled Fed/FDIC difference at 1970, and a further 9437 banks that are created as new Banks during the period from 1970 to 2013, and hence have zero ancestors); the

Supplementary Table 1: US Banking population using data taken from the 21st to 57th Annual Reports of the Board of Governors of the Federal Reserve System [1]

Real data populations	Count
Initial Bank population in 1934	15848
New Banks (including revision[a])	3789
Mergers/Liquidations	5479
Final Bank population in 1969	14158
**Model input**	
Initial Bank population in 1934	19637
Final Bank population in 1969	14158

[a] Note: In 1947 there was a revision resulting in an adjustment in the inventory of c. 115 banks. We assumed those to be "new banks".

remaining 14158 banks have their ancestries assigned based on simulations modelling the period from 1934 to the end of 1969.

Supplementary Table 2 shows the population at the end of 1969 that results from the simulations. These are averaged results and the average, maximum ancestry at the end of 1969 is around 37, which is very small relative to the total population of 27631 banks. We verified through simulation running from 1970 to 2013 that the ancestry distributions which result are insensitive to this change, as might be expected given its limited size. We therefore conclude that the results of the main paper are not strongly affected by the chosen initial condition, i.e. setting all banks to have zero ancestors.

II. SUPPLEMENTARY REFERENCES

[1] `http://fraser.stlouisfed.org/publication/?pid=117`, [Online; accessed 25-March-2014].

Supplementary Table 2: Synthetic 1970 US Banking population initial condition: simulated data for evolution from 1934 to 1969

Ancestry values	Count
1	11091
2	1919
3	611
4	251
5	120
6	63
7	36
8	21
9	14
10	8
11	6
12	4
13	3
14	2
15	1
16	1
17	1
19	1
21	1
37	1
